# Supplementary material for: PARP10 Multi-Site Auto- and Histone MARylation Visualized by Acid-Urea Gel Electrophoresis
Source: Cells. 2021 Mar 15;10(3):654. doi: 10.3390/cells10030654 (PMC7998796; doi:10.3390/cells10030654)
Supplement: Supplementary file 1 [file cells-10-00654-s001.pdf]

## Supplementary Materials accompanying

# PARP10 Multi-site Auto- and Histone MARylation Visualized by Acid-Urea Gel Electrophoresis

**Antonio Ginés García-Saura[1] and Herwig Schüler[1,2,*]**

[1] Department of Biosciences and Nutrition, Karolinska Institutet, Huddinge, Sweden
[2] Center for Molecular Protein Science, Department of Chemistry, Lund University, Lund, Sweden
* Correspondence: herwig.schuler@biochemistry.lu.se; Tel.: +46-46-222-7582

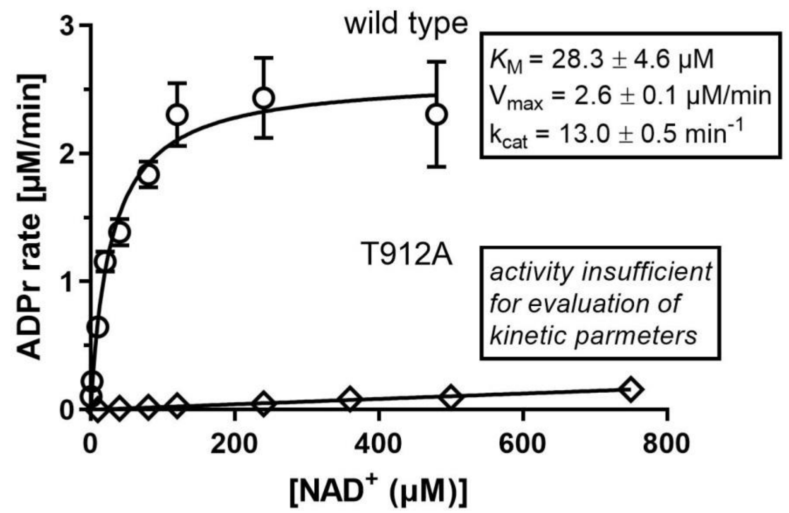


**Supplementary Figure 1. The T912A mutation abolishes PARP10 auto-MARylation activity.** Kinetic analysis of the auto-MARylation activity of wild type and T912A PARP10 (catalytic domain construct). Activity was assessed using 2% $N^6$-biotin-NAD$^+$ as substrate, and signals were quantified using biotinylated GRB14 protein as described in detail before. [1]

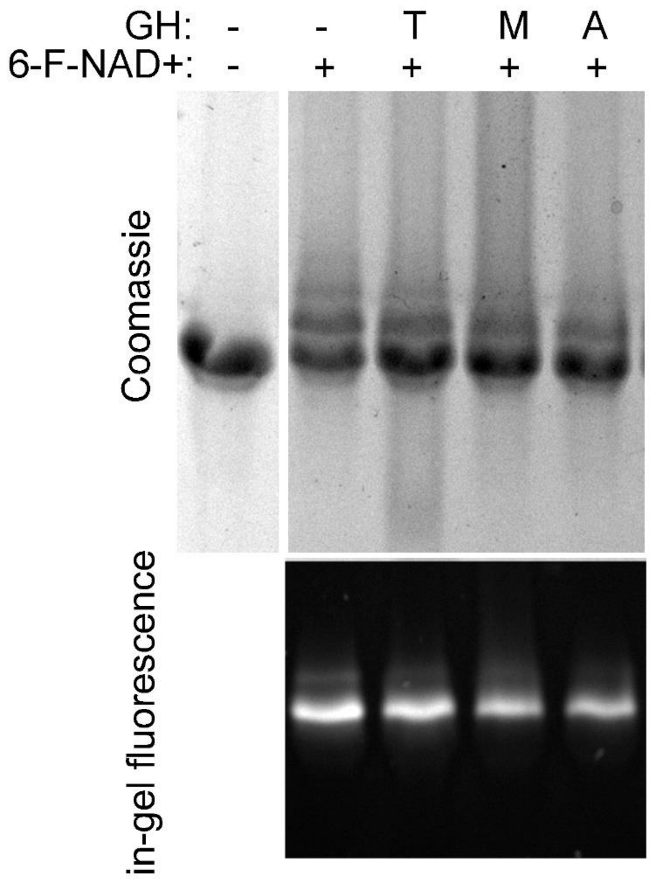


**Supplementary Figure 2. TARG, MacroD2 and ARH3 appear unable to remove 6-fluoro-ADP-ribosyl groups.** PARP10 catalytic domain was incubated with 1 mM 6-F-NAD$^+$ for 1 h at room temperature to allow auto-MARylation. Then, aliquots of the reaction were incubated with TARG (T), MacroD2 (D) or ARH3 (A) and incubated for 1 h at room temperature, as in figure 2 of the main text. Reactions were separated by AU-PAGE.

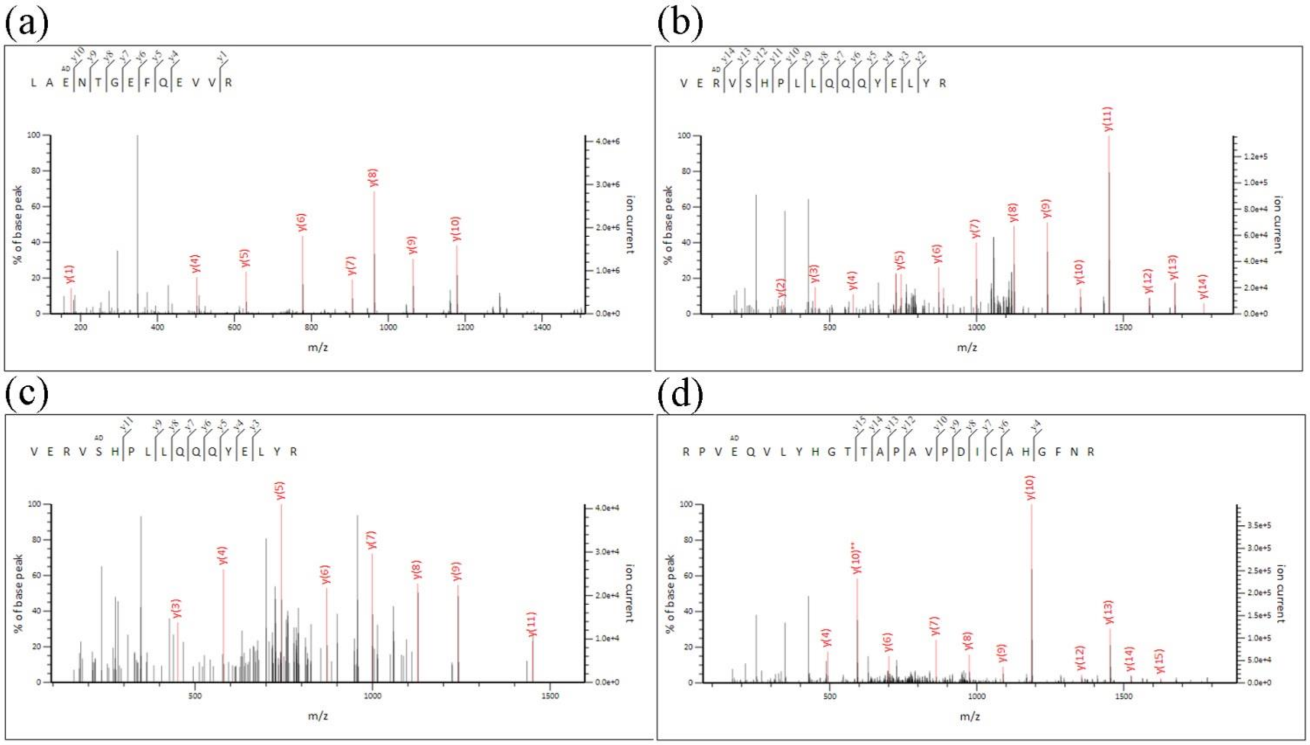


**Supplementary Figure 3. Examples of mass spectra of ADP-ribosyl-peptides listed in Table 1 of the main text.** (**a**) E825: LAENTGEFQEVVR, E3-ADP-ribosyl (678.2747 Da, [M+3H]$^{3+}$); (**b**) R855: VERVSHPLLQQQYELYR, R3-ADP-ribosyl (678.2747 Da, [M+3H]$^{3+}$); (**c**) S857; VERVSHPLLQQQYELYR, S5-ADP-ribosyl (675.5562 Da, [M+4H]$^{4+}$); (**d**) E822: RPVEQVLYHGTTAPAVPDICAHGFNR, E4-ADP-ribosyl (862.3841 Da, [M+4H]$^{4+}$).

**Reference**

1. Thorsell, A.G.; Ekblad, T.; Karlberg, T.; Löw, M.; Pinto, A.F.; Trésaugues, L.; Moche, M.; Cohen, M.S.; Schüler, H. Structural Basis for Potency and Promiscuity in Poly(ADP-ribose) Polymerase (PARP) and Tankyrase Inhibitors. *Journal of medicinal chemistry* **2017**, *60*, 1262-1271, doi:10.1021/acs.jmedchem.6b00990.
